# Intestinal Permeation Characteristics via Non-Everted Gut Sac of Diterpene Lactones from Pure Andrographolide and Three Different Andrographis Extracts: An Investigation into Liqui-Mass with Different Solvents

**DOI:** 10.3390/pharmaceutics18010090

**Published:** 2026-01-10

**Authors:** Peera Tabboon, Ekapol Limpongsa, Thitiphorn Rongthong, Thaned Pongjanyakul, Napaphak Jaipakdee

**Affiliations:** 1 Division of Pharmaceutical Technology, Faculty of Pharmaceutical Sciences, Khon Kaen University, Khon Kaen 40002, Thailand; peerata@kku.ac.th (P.T.); thaned@kku.ac.th (T.P.); 2 Center for Research and Development of Herbal Health Products, Khon Kaen University, Khon Kaen 40002, Thailand; 3 College of Pharmacy, Rangsit University, Rangsit 12000, Thailand; ekapol.l@rsu.ac.th; 4 Chulabhorn Royal Pharmaceutical Manufacturing Facilities, Chulabhorn Royal Academy, Chon Buri 20180, Thailand; thitiphorn.ron@cra.ac.th

**Keywords:** *Andrographis* extract, andrographolide, 14-deoxy-11,12-didehydroandrographolide, diterpene lactones, liquid solvents, intestinal absorption

## Abstract

**Objectives**: This study aimed to assess the intestinal permeation behaviors of andrographolide (AG) and 14-deoxy-11,12-didehydroandrographolide (DDAG), diterpene lactones from *Andrographis paniculata* extract (APE), pure AG, and three distinct source APEs. The effects of different solvents were also investigated. **Methods**: Solubility investigation was performed using APE. APEs and pure AG were prepared as liqui-masses, cohesive mixtures of APE, solvents, and solid carriers. PXRD, in vitro release, and ex vivo intestinal permeation using the non-everted gut sac method were investigated. **Results**: Solubility of AG and DDAG in N-methyl-2-pyrrolidone (NMP) > NMP/diethylene glycol monoethyl ether (DG) mixtures > DG. PXRD indicated that crystallinity loss of liqui-mass was affected by solvent’s solvency capacity. The release behaviors of AG and DDAG in phosphate buffer from pure AG and APEs varied depending on their solid state. The release efficiencies of AG and DDAG from liqui-mass systems increased significantly. The apparent permeability (*P_app_*) of AG from pure AG was 0.11 ± 0.05 ×10^−5^ cm·s^−1^, which was 11–25 times less than that of APEs. The *P_app_* of DDAG from various APEs was comparable, ranging between 5.95 and 7.37 × 10^−5^ cm·s^−1^. The presence of a solvent, specifically NMP, in liqui-mass significantly enhanced the release rate and permeation flux. The *P_app_* of AG and DDAG from liqui-mass increased by factors of 1.0–2.3 and 1.1–2.7, respectively. **Conclusions**: This study is the first to emphasize the differences in the release and intestinal permeation characteristics of AG and DDAG from APEs. These findings offer essential insights into the intestinal permeation behavior of diterpene lactones, along with a straightforward mechanistic strategy for enhancement.

## 1. Introduction

The utilization of herbal medicinal products and phytopharmaceuticals is experiencing significant global growth as a primary component of healthcare and traditional medicine. The sustained popularity of herbal medication products can be attributed to their perceived natural origin and historical usage, which are readily accessible at a price that is affordable for the average consumer [1]. Although many medicinal herbal compounds exhibit significant therapeutic potential, their clinical application is often hampered by challenges related to their inadequate oral bioavailability. This is due to their unfavorable physicochemical characteristics, such as hydrophobicity and poor water solubility. To overcome this challenge, various pharmaceutical approaches have been employed, resulting in promising results [2,3,4,5,6,7].

*Andrographis paniculata* (Burm. f.) Nees (*A. paniculata*), a flowering plant belonging to the Acanthaceae family, is widely used as a herbal remedy in traditional medicine, including Ayurveda, Chinese, and Thai practices, for treating various illnesses. *A. paniculata* exhibits a diverse range of pharmacological effects, including analgesic, anticancer, antidiabetic, anti-inflammatory, antimalarial, antimicrobial, antipyretic, antiretroviral, and immunomodulatory effects [8,9,10,11,12,13,14]. *A. paniculata* contains several active principles, primarily ent-labdane diterpenoids, flavonoids, and polyphenols [8,14,15]. Two primary diterpene lactone constituents responsible for the pharmacological effects of *A. paniculata* are andrographolide (AG) and 14-deoxy-11,12-didehydroandrographolide (DDAG) [10,11,15,16].

**Figure 1:**
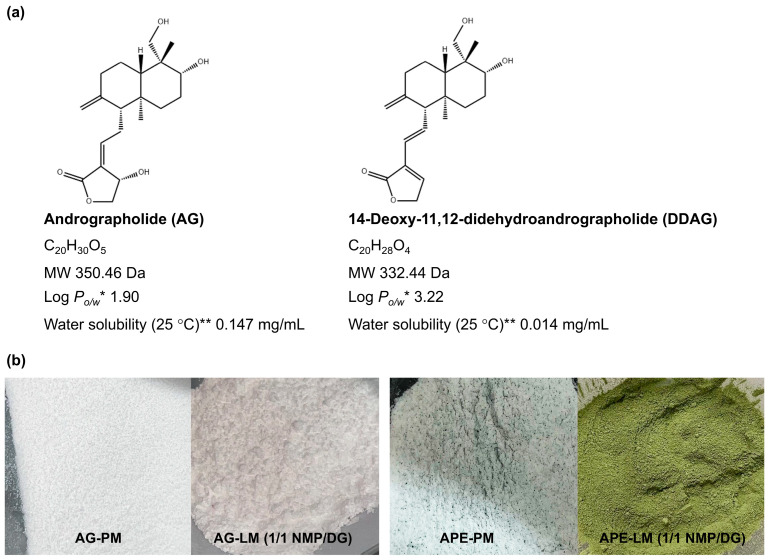
(**a**) Chemical structures and physicochemical properties of andrographolide (AG) and 14-deoxy-11,12-didehydroandrographolide (DDAG). * Log *P_o/w_*, log octanol-water partition coefficient, ** Water solubility at 25 °C estimated from Log *P_o/w_*. (**b**) Appearances of AG-PM, AG-LM, APE-PM, and APE-LM.

AG and DDAG are hydrophobic phytopharmaceuticals with predicted log octanol-water partition coefficients (Log *P_o/w_*) of 1.90 and 3.22, respectively. Both compounds have negligible solubility in water, as shown in Figure 1. The inadequate water solubility of AG and DDAG is one of the primary factors contributing to their very low absolute bioavailability (<3.5 %) [17,18,19]. As is known, the aqueous solubility of active pharmaceutical substances has consistently posed a challenge because insufficient aqueous solubility can restrict the bioavailability of oral formulations. Aqueous solubility and drug permeability are the two primary characteristics associated with oral bioavailability, along with other factors such as first-pass metabolism and susceptibility to efflux mechanisms [20]. Additionally, a notable discrepancy in the dissolution rate and quantity of diterpene lactones, including AG, DDAG, neoandrographolide (NAG), and 14-deoxyandrographolide, from various sources across test media has been demonstrated [21]. The constrained and fluctuating dissolution attributes of diterpene lactones, especially AG, may lead to irregular bioavailability, presenting considerable obstacles in attaining consistent therapeutic values [14,21].

Various pharmaceutical techniques, including liquisolid technology [22,23], self-emulsifying, and lipid-based systems, have been successfully employed to enhance the dissolution behavior and, consequently, the bioavailability of AG and APE [7,22,23,24,25,26,27]. These drug delivery systems typically consist of a liquid solvent as a fundamental component. The liquid solvents in these systems not only function directly as solvents for active compounds to enhance dissolution, but may also act as permeation-modifying additives for compounds with limited permeability [22,28,29,30]. Although *A. paniculata* is a well-known and extensively studied plant species, studies on the impact of liquid solvents on the intestinal permeation of diterpene lactones are scarce. Furthermore, studies on the intestinal permeation characteristics of diterpene lactones derived from *A. paniculata* extract (APE) are limited. No studies have addressed the intestinal permeation behavior of APE from various sources.

The current investigation aimed to evaluate the intestinal permeation behaviors and parameters of AG and DDAG, which are phytopharmaceutical diterpene lactone markers of APE, using a non-everted ex vivo intestinal sac permeation model. Three distinct source APEs, each with varying AG and DDAG contents, were utilized as sources of AG and DDAG. A liqui-mass, which is a cohesive and non-flowable mixture of APE, liquid solvents, and a solid carrier (Neusilin and colloidal silica (CSD) mixture), was developed. N-Methyl-2-pyrrolidone (NMP), diethylene glycol monoethyl ether (DG), and NMP/DG mixtures were investigated as liquid solvents in the liqui-mass. A liqui-mass of pure AG was fabricated and evaluated for comparison. Liqui-mass serves as an intermediary phase in powdered solution technology, which can subsequently be converted into a dry and free-flowing liquisolid form or into micron-sized particles (liqui-pellet form). Liqui-mass technology has been implemented owing to its efficacy in dissolution enhancement, straightforward manufacturing process, cost-effectiveness, environmentally sustainable technology, potential for large-scale production, and adaptability to formulation modifications [31,32,33]. This study presents several original findings on various aspects. This study is the first to report the application of liqui-mass technology for the development of oral dissolution enhancement systems for AG and APEs. The release of diterpene lactones in phosphate buffer and their intestinal permeation behaviors were systematically characterized for the first time, with an emphasis on the influence of the liquid solvents. The variation in the intestinal permeation behavior of diterpene lactones from APE obtained from different sources was also revealed. A correlation between the presence of DDAG with different contents in the extract matrix and the permeability of AG molecules was also identified for the first time.

## 2. Materials and Methods

### 2.1. Materials

#### 2.1.1. Plant Material and Extract

**Figure 2:**
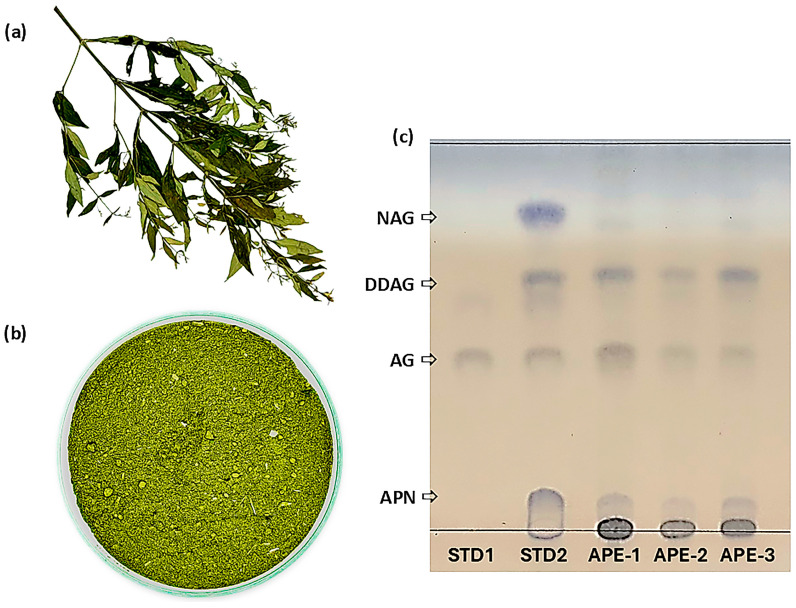
(**a**) Dried aerial parts of *A. paniculata*, (**b**) finely powdered dried leaves of *A. paniculata* after grinding, and (**c**) TLC profiles of reference standard 1 (STD1, AG solution), reference standard 2 (STD2, solution mixture of AG, NAG, DDAG, and andrograpanin (APN), APE-1, APE-2, and APE-3.

Three ethanolic crude extracts of *A. paniculata* (Burm. f.) Wall. ex Nees (dried leaves), designated APE, were prepared using Soxhlet extraction. *A. paniculata* specimens were authenticated by a botanist, and their associated herbarium voucher collections (herbarium voucher numbers: HB 200/66-HB 202/66) were maintained at the Center for Research and Development of Herbal Health Products, Khon Kaen University, Thailand. The physical features of the dried aerial parts of *A. paniculata* and its finely powdered dried leaves are shown in Figure 2a and Figure 2b, respectively.

APE-1 was derived from *A. paniculata* sourced from the community enterprise of local herbal cultivators and processors in Kalasin Province, Thailand (herbarium voucher number: HB 202/66). APE-1 comprised 24.1 ± 1.1% AG and 4.1 ± 0.1% DDAG. APE-2 was derived from *A. paniculata* sourced from the Ban Dong Bang Herbal Community in Prachinburi Province, Thailand (herbarium voucher number: HB 200/66). APE-2 comprised 17.1 ± 1.4% AG and 5.8 ± 0.1% DDAG. APE-3 was derived from *A. paniculata* sourced from the Diamond Phu Phan Sacha Inchi Product Group in Sakon Nakhon Province, Thailand (herbarium voucher number: HB 201/66). APE-3 comprised 14.9 ± 1.2% AG and 13.8 ± 1.2% DDAG. Powdered *A. paniculata* was also identified in accordance with the USP-NF monograph for Powdered Andrographis [34] using thin-layer chromatographic (TLC) techniques. The TLC profiles of all three extracts (APE-1, APE-2, and APE-3) displayed distinct bands that aligned with the reference standards (AG, NAG, DDAG, and andrograpanin (APN)), confirming adherence to the pharmacopeial identification criteria (Figure 2c).

#### 2.1.2. Chemicals and Solvents

Andrographolide (pure AG, ≥98% purity), andrograpanin (APN, 99.39% purity), neoandrographolide (NAG, 98.84% purity), and 14-deoxy-11,12-didehydroandrographolide (DDAG, ≥98% purity) were obtained from Weifang Wehibest Supply Chain Co., Ltd. (Weifang, China). The reference standard AG (99.67% purity) was purchased from the Bureau of Drug and Narcotic Department of Medical Sciences, Ministry of Public Health (Nonthaburi, Thailand). N-methyl-2-pyrrolidone (NMP) was obtained from QRëC (New Zealand). Diethylene glycol monoethyl ether (DG, Transcutol^®^ HP, Gattefossé SAS, Saint-Priest, France) was sourced by Rama Production, Co., Ltd., Bangkok, Thailand. Neusilin^®^ US2 (Neusilin), or magnesium aluminometasilicate, was purchased from Fuji Chemical Industries Co., Ltd. (Osaka-shi, Osaka, Japan). Colloidal silicon dioxide (CSD, Aerosil^®^ 200) was purchased from Maxway Co, Ltd. (Prawet, Bangkok, Thailand). Absolute ethanol (EtOH, L Pure^®^ 99.8) was obtained from the Liquor Distillery Organization (Chachoengsao, Thailand). Hydrochloric acid (HCl) was sourced from RCI Labscan Co., Ltd. (Pathumwan, Bangkok, Thailand). HPLC-grade methanol and acetonitrile were purchased from Fisher Scientific (Loughborough, UK). Potassium dihydrogen phosphate was obtained from Fisher Scientific (Waltham, MA, USA). Sodium hydroxide was purchased from Ajax Finechem Pty Ltd. (Taren Point, New South Wales, Australia). Sodium chloride, disodium hydrogen phosphate, magnesium chloride, potassium chloride, and sodium dihydrogen phosphate were obtained from KEMAUSChemicals (Cherrybrook, New South Wales, Australia). All compounds were used as obtained, without additional purification.

#### 2.1.3. Animals

Male Wistar rats aged 6–8 weeks and weighing 200–250 g were procured from Nomura Siam International Co., Ltd. (Pathumwan, Bangkok, Thailand). The animals were housed at the Northeast Laboratory Animal Center at Khon Kaen University in Khon Kaen, Thailand, under standard conditions for seven days prior to the initiation of the investigation. The temperature in the animal chamber was maintained between 21–25 °C, with humidity levels fluctuating between 30% and 60% relative humidity (RH). A normal diet and water were provided ad libitum. The conditions followed a 12-h cycle of illumination and obscurity. The experimental methods were evaluated and approved by the Institutional Animal Care and Use Committee of Khon Kaen University (IACUC KKU) (Khon Kaen, Thailand). The protocol certificate number is officially recorded as IACUC-KKU-15/67.

### 2.2. AG and DDAG Solubility

The solubilities were evaluated using the shake-flask methodology [33,35]. An excess APE-3 powder was combined with 1 g of each tested liquid in sealed tubes. The resultant products underwent ultrasonic treatment for 60 min and were subsequently maintained in a thermostatic shaking water bath for 30 h at 30 ± 1 °C for nonvolatile liquid solvents and at 37 ± 1 °C for aqueous media. The obtained supersaturated mixtures were subsequently processed using a 0.45 μm nylon membrane syringe filter (Agilent Technologies, Inc., Santa Clara, CA, USA) and diluted with methanol. The resulting solution was analyzed for AG and DDAG content using HPLC.

### 2.3. Preparation of Physical Mixture and Liqui-Mass

Four unique liqui-mass preparations of AG (AG-LM) and APEs (APE-LM) were developed using different liquid solvents in addition to physical mixtures (AG-PM and APE-PM) (Table 1). In summary, the requisite quantity of AG or APE was determined and dissolved in a particular quantity of liquid solvent with the assistance of an equal quantity of EtOH. The mixture was ultrasonicated for 30 min. The resultant solution was blended with the Neusilin-CSD mixture. The blend was periodically stirred to promote the evaporation of EtOH at ambient temperature for 4 h. Residual EtOH was removed by oven-drying at 45 °C for 4 h until a consistent weight was achieved. For the physical mixture (PM) admixture, the requisite quantity of AG or APE was measured and geometrically blended with Neusilin-CSD using a rotomixer for 5 min. All manufactured mixtures were stored in a tightly closed, light-resistant pouch and kept under refrigerated conditions until use.

### 2.4. Content Determination

The quantities of AG and DDAG in the tested specimens, corresponding to 3 mg of AG, were measured and dissolved in 100 mL of EtOH in a volumetric flask. The mixture was ultrasonicated for 60 min and magnetically stirred for 20 h. The solution was subjected to 0.45 μm filtration, and the amounts of AG and DDAG were assessed using HPLC.

### 2.5. Solid-State Investigation

Powder X-ray diffraction (PXRD) was performed using a Rigaku MiniFlex powder X-ray diffractometer (Rigaku Holdings Corporation, Tokyo, Japan). The measurements were conducted using a Cu Kα source (λ = 1.54 Å) produced at 40 kV and 15 mA with a Bragg angle 2θ ranging from 5° to 35° and a step angle of 0.01° (2θ) per second.

OriginPro software (OriginLab Corporation, Northampton, MA, USA) was used to assess the crystallinity index (CI) [36]. The integrated area beneath the crystallized peak of the XRD diffractogram was determined, and the CI was calculated using Equation (1). The percentage of crystallinity reduction of the liqui-mass formulation relative to that of the physical mixture was determined using Equation (2).

*CI* = (*Area of crystalline peaks/Total area under XRD pattern*) × 100 (1)

*Percentage of crystallinity reduction* = ((*CI_PM_ – CI_LM_*)/*CI_PM_*) × 100 (2)

where *CI_PM_* signifies the CI of the physical mixture (AG-PM and APE-PM), and *CI_PM_* denotes the CI of the liqui-mass (AG-LM and APE-LM).

### 2.6. In Vitro Release

A double-walled jacketed beaker containing a magnetic bar was filled with 100 mL of phosphate buffer, which served as the receiver medium, and the hydrodynamics were maintained by stirring at a rate of 200 rpm (WH-610D magnetic stirrer, Wiggens GmbH, Wuppertal, Germany). The temperature was maintained at 37 ± 0.5 °C (WiseCircu WCB-6 model; Daihan Scientific Co., Ltd., Seoul, Republic of Korea). To initiate the experiment, PM or liqui-mass powder of pure AG or APEs, corresponding to 10 mg of AG, was directly introduced into the medium in the beakers. At designated times, a 1 mL aliquot of the media was extracted and replaced with 1 mL of pre-heated media. The collected samples underwent 0.45 μm filtration before assessing AG and DDAG content using HPLC.

### 2.7. Ex Vivo Intestinal Permeation

#### 2.7.1. Isolation of Intestinal Segments

An intestinal segment from male Wistar rats was used to assess transport through the intestinal membrane, as described previously [29,33,37]. Following a 12-h fast, the animals were euthanized via intraperitoneal administration of thiopental sodium (120 mg/kg) on the experimental date. The abdomen was surgically accessed, and a segment of the intestine (duodenum-ileum) was excised and meticulously washed and cleaned with oxygenated Krebs-Ringer solution to remove the solid mass. The purified intestine was then divided into segments with a mean length of 24 ± 6 cm (*n* = 108). The circumference of each intestinal lumen was assessed at three distinct spots, and the mean value was calculated.

#### 2.7.2. Permeability Study

Permeability behavior was investigated using non-everted gut sac methodologies [29,33,37]. The terminally tied intestinal lumen was filled with a perfusion mixture comprising the tested formulation (AG-PM, APE-PM, AG-LM, or APE-LM corresponding to 20 mg of AG) in a pH 6.8 phosphate buffer (6.0 mL/g for PM and 3.5 mL/g for LM powders). The dimensions of the sac that interacted with the tested admixture and end-to-end ties were accurately measured and recorded. The resultant sacs were positioned in a glass vessel filled with 100 mL of pH 7 phosphate-buffered saline (PBS) and maintained at 37 °C with continuous aeration (95% O_2_ plus 5% CO_2_). At specified periods, a 1 mL aliquot of PBS was extracted and replaced with 1 mL of PBS. The obtained sample was subjected to 0.45 μm filtration before evaluating the AG and DDAG levels using HPLC.

#### 2.7.3. Data Assessment

The cumulative permeation amount of AG or DDAG per unit area (*Q_P_*) was measured and plotted over time. The permeation flux in the steady-state region (*J_ss_*) was calculated by computing the slope of the linear segment of the curve. The apparent permeability (*P_app_*) from the mucosal-to-serosal side (M⟶S) was calculated by dividing the flux by the initial concentration (*C*_0_) of AG or DDAG in the sac, as shown in Equation (3). The permeability enhancement ratio (*ER*) was calculated using Equation (4).

*J_ss_* = *dQ*/*dt∙*1/*A* = *P_app_∙C*_0_ (3)

where *dQ*/*dt* signifies the permeation rate of AG or DDAG, and *A* defines the surface area of the gut segment in contact with the examined powder.

*ER* = *P_app_ of LM*/*P_app_ of PM* (4)

### 2.8. HPLC Method for AG Determination

AG and DDAG were quantified using an LC-2050 series Shimadzu HPLC system paired with an autosampler and a diode array detector set at 254 nm [38]. Chromatographic separation was performed using a ZORBAX reversed-phase column (Eclipse Plus C18, 4.6 × 150 mm, 3.5 µm, Agilent Technologies, Inc., USA) maintained at 20 °C. The mobile phase comprised 0.1% formic acid in acetonitrile solution (A) and 0.1% formic acid in an aqueous solution (B). Gradient program measurements were conducted at a flow rate of 1.4 mL/min under the following conditions: 0–3 min, isocratic maintained at 30% A; 3–6 min, gradient to 40% A; 6–9 min, isocratic maintained at 40% A; 9–10 min, gradient to 80% A; 10–12 min, isocratic maintained at 80% A; 12–13 min, gradient to 30% A; 13–15 min, isocratic maintained at 30% A. For content and dissolution analyses, the injection volume was 20 µL, whereas 40 µL was used for intestinal permeation determination. Chromatographic analysis was conducted using the Shimadzu Class VP version 5.124 software (Shimadzu Corporation, Kyoto, Japan).

### 2.9. Statistical Evaluation

The results are presented as the mean ± standard deviation (SD). Statistical assessment of all results was performed using one-way analysis of variance in SPSS software for MS Windows, version 19 (SPSS (Thailand) Co., Ltd., Bangkok, Thailand). The significance of the mean difference was assessed using Tukey’s test, with a threshold for significance set at *p* < 0.05.

## 3. Results

### 3.1. Solubility of AG and DDAG

The solubilities (30 °C) were studied using APE-3, which encompassed 14.9% AG and 13.8% DDAG (1.08:1 AG:DDAG ratio), as the source of AG and DDAG. The high solubility of APE-3 in NMP and 1:1 NMP/DG, with 1 g of APE-3 dissolved in less than 1 g of liquid, makes it impossible to attain a saturated state of APE-3 in these solvents. The solubility of AG in these solvents is expected to exceed 9.65% and 9.09%, respectively. DG yielded only 5.13% AG solubility. The incorporation of NMP boosted the AG solubility in DG, with the value increasing proportionally to the NMP ratio.

The limited solubility of the AG compounds is consistent with a previous report [22]. According to this report, pure AG exhibited restricted solubility of ≤1% *w*/*w* in a wide range of pharmaceutical liquids, including DG, while demonstrating good solubility of 26% *w*/*w* in NMP at 25 °C. Interestingly, the solubility of DDAG was lower than that of AG. The solubility of DDAG was approximately 5% *w*/*w*, regardless of the solvent type. This may be related to the greater hydrophobicity of DDAG compared with that of AG molecules. Nonetheless, there is a significant lack of data on the solubility of DDAG compounds.

**Figure 3:**
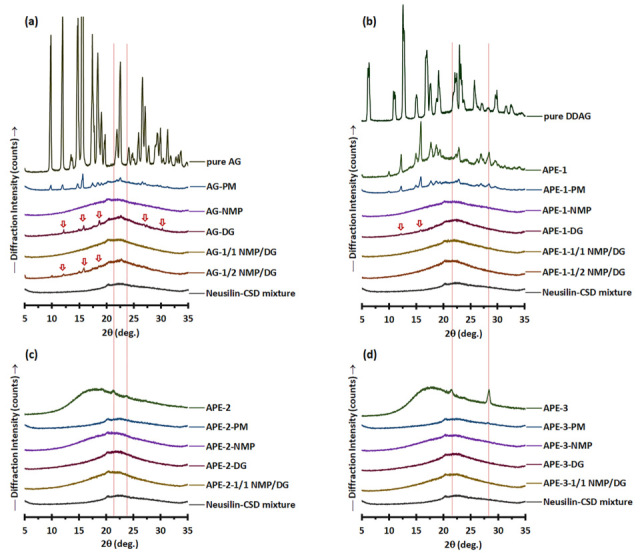
PXRD patterns of (**a**) pure AG, AG-PM vs. AG-LM; (**b**) pure DDAG, APE-1, APE-1-PM vs. APE-1-LM; (**c**) APE-2, APE-2-PM vs. APE-2-LM; and (**d**) APE-3, APE-3-PM vs. APE-3-LM. The arrows denote the crystalline peaks of AG.

The solubilities of AG and DDAG in aqueous media at physiological temperature (37 °C) were also examined (Table 2). AG from APE-3 was soluble in DI water, 0.1 M HCl, and pH 6.8 phosphate buffer at concentrations of 0.24–0.30 mg/mL. The solubility values of DDAG were generally lower than those of AG molecules, regardless of the liquid type. This is clearly related to the greater hydrophobicity of the DDAG molecule. In comparison to the reported AG solubility derived from pure AG, the AG solubility value obtained from APE-3 in this study was higher. A recent study indicated that the saturation concentrations of AG at 37 °C in pure water, pH 1.2 HCl, and pH 6.8 PBS were approximately 0.041, 0.039, and 0.033 mg/mL, respectively [39]. This could be associated with the relatively amorphous structure of the AG compound in APE-3 (Figure 3d) compared to that of pure AG compound (Figure 3a). The apparent solubility of a compound in its crystalline form is limited compared to that of its amorphous form, which is present under conditions of elevated free energy [40,41].

### 3.2. Liqui-Mass and Physical Mixture Powder of AG and DDAG

#### 3.2.1. Physical Appearance and AG Content

The liqui-mass powder, produced by loading the AG-nonvolatile liquid solvent mixture (1 g) onto the Neusilin-CSD powder (1 g), was a relatively cohesive and non-flowable powder (Figure 1b). A liquid loading parameter of 1.0 was used to ensure that the quantity of liquid in all the formulations remained consistent. The AG-based powder was white, whereas the APE-based powder was homogeneous and green.

The actual AG content of the AG-LM formulations ranged from 98.9% to 101.9% of the theoretical loading of 5% (Table 3). The formulated APE-LM demonstrated theoretical AG and DDAG loading percentages ranging between 1.49–2.41% and 0.41–1.38%, respectively, depending on the AG and DDAG present in the APE. The actual AG and DDAG contents of the APE-LM formulations varied from 96.5% to 101.9% of their theoretical loadings. The low SD values of the actual AG and DDAG contents indicate a considerably lower content variability in APE-LMs. The uniformity of the AG and DDAG contents in the APE-LM formulations is related to the process of dissolving or dispersing APE in a liquid medium prior to its application to the broad surface area of the carrier-coating systems. For the PM formulations, the significant variation in the SD values indicated a lack of uniformity in the powder mixture. These were correlated with the inhomogeneous color of the APE in the powder mixture (Figure 1b).

#### 3.2.2. Solid State Analysis

The PXRD patterns of the powder mixture of pure AG (Figure 3a) revealed that AG-PM displayed the distinct characteristics of pure AG within the range of 9.8–22.6° 2θ, in addition to the amorphous halo pattern associated with Neusilin-CSD. Diffraction peaks corresponding to those of pure AG were also observed in the AG-DG and AG-1/2 NMP/DG liqui-mass, albeit with diminished peak intensity and a reduced number of peaks compared to those of AG-PM. This indicates a partial loss of crystalline peaks and the formation of a molecularly dispersed or amorphous solid of AG molecules in the AG-DG and AG-1/2 NMP/DG liqui-mass systems. The percentage of crystallinity reduction of the AG-DG and AG-1/2 NMP/DG liqui-mass powders was 49% and 67%, respectively, compared to that of AG-PM. Conversely, the PXRD patterns of AG-NMP and AG-1/1 NMP/DG liqui-mass exhibited only a halo pattern similar to that of Neusilin-CSD, signifying the amorphous or dissolved state of AG. The percentage of crystallinity reduction of the AG-NMP and AG-1/1 NMP/DG liqui-mass formulations was 100%.

The PXRD pattern of APE-1-PM (Figure 3b) reveals notable features of APE-1, characterized by prominent diffraction peaks at 2θ of 12.0–22.8°, 26.4°, and 28.2°, alongside an amorphous halo pattern linked to Neusilin-CSD. APE-1 displayed diffraction peaks that aligned with those of pure AG [22,25,42,43], suggesting that the AG molecule in APE-1 exists in a crystalline form. It is recognized that crystalline materials exhibit distinct, well-defined peaks, in contrast to amorphous materials, which, owing to their absence of long-range order, produce broader features that lack the clarity of specific forms [44]. The diffraction peak observed at 28.4° 2θ for APE-1 coincided with that of DDAG. Pure DDAG showed diffraction peaks at 6.3°, 10.8°, 12.6°, 15°, 16.9°, 19.1°, 22.9°, 25.7°, 28.3°, 29.8°, 32.4°, and 36.4° 2θ. Notably, the PXRD pattern of DDAG has not been previously documented.

All APE-1-LM samples exhibited halo-like PXRD patterns. However, in the case of APE-1-DG, minor peaks at 12.3° and 15.9° 2θ, suggestive of APE-1 crystalline features, were still observed. The crystallinity reduction of APE-1-DG liqui-mass was measured to be 85%. APE-1-1/2 NMP/DG exhibited a crystallinity reduction of 94%, whereas APE-1-NMP and APE-1-1/1 NMP/DG showed complete crystallinity reduction of 100%. This indicates incomplete molecular dispersion or amorphous solid formation of AG molecules in APE-1-DG and APE-1-1/2 NMP/DG systems. The crystallinity patterns of AG in the APE-1-LM systems, analogous to those of AG-LM, are substantially correlated with the solvency capacity of the solvent for AG and DDAG molecules, leading to the molecularly dispersed or amorphous solid formation of these components. The theoretical loading of AG in APE-1-LM (2.41%) was approximately half that of AG-LM (5.0%). Furthermore, APE-1-LM exhibited minimal DDAG concentrations, which could be attributed to the low DDAG levels of APE-1. The reduced concentration of APE constituents, particularly AG and DDAG, may facilitate their transformation into molecularly dispersed or amorphous solid formations, facilitated by the liquid solvent in the liqui-mass systems.

Although the minute peak observed for APE-2-PM and APE-3-PM suggested that the crystalline compounds of APE-2 and APE-3 could be identified from their PM, all formulations of APE-2-LM (Figure 3c) and APE-3-LM (Figure 3d) exhibited broad PXRD patterns. All APE-2-LM and APE-3-LM formulations demonstrated complete crystallinity reduction compared to the physical mixture of 100%. These findings indicate that all the solvents utilized in the examined liqui-mass were adequate for solubilizing the crystalline compounds in these two APEs. Notably, APE-2 and APE-3 exhibited broad PXRD patterns with no evidence of AG crystalline peaks, whereas APE-3 indicated the presence of crystalline DDAG.

#### 3.2.3. In Vitro Release Behaviors and Parameters

Release studies were conducted under nonsink conditions using a phosphate medium. This study aimed to explore the release characteristics of AG and DDAG from liqui-mass formulations under intestinal conditions that mimic those employed in intestinal permeation investigations. It should be noted that the dissolution methods employed under nonsink conditions are crucial for assessing the efficacy of supersaturated formulations and preparations. The nonsink conditions exhibited the most gradual dissolution processes and demonstrated some of the most varied dissolution profiles, suggesting a greater capacity for discrimination than sink conditions [45,46].

**Figure 4:**
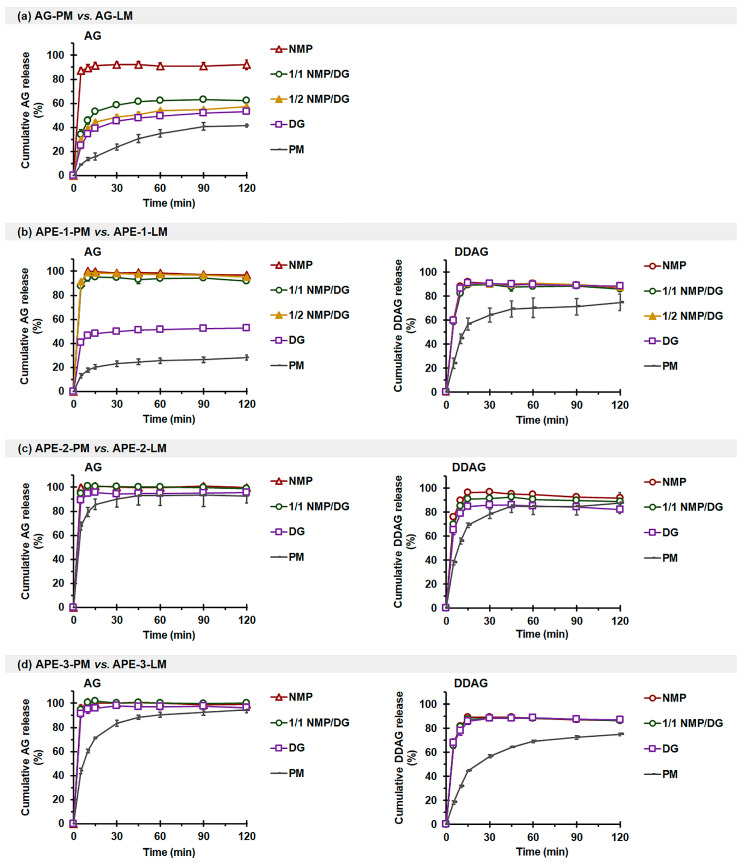
Effects of solvents on the in vitro release profiles of AG (**left**) and DDAG (**right**) in phosphate buffer: (**a**) AG-PM vs. AG-LM, (**b**) APE-1-PM vs. APE-1-LM, (**c**) APE-2-PM vs. APE-2-LM, and (**d**) APE-3-PM vs. APE-3-LM. Each value represents the mean ± SD of *n* = 3.

As illustrated in Figure 4a and Table 4, the release of AG from AG-PM was limited. AG-LM demonstrated a notable enhancement in AG release, encompassing both the initial release rate (Q_R-5min-AG_) and efficiency (RE_120min-AG_) parameters. The extent of AG-LM release enhancement was correlated with the solvent system’s solvency capacity and the disappearance of AG crystallinity in LM formulations. For AG-NMP, the release of AG was almost complete, with RE_120min-AG_ reaching 87.0%. The AG-1/1 NMP/DG, AG-1/2 NMP/DG, and AG-DG systems exhibited increases of 3.8-fold, 3.3-fold, and 2.8-fold, respectively, in Q_R-5min-AG_ and enhancements of 1.5 to 1.9 times in RE_120min-AG_ in comparison to AG-PM. The poor release rate and efficiency of AG in the phosphate buffer medium under nonsink conditions are consistent with previous studies [47]. This is related to the poor solubility of AG compounds in the pH 6.8 phosphate buffer medium, identical to that observed in water and pH 1.2 HCl medium [39,47].

The enhancement of AG release from AG-LM correlates with the molecularly dispersed or amorphous solid formation of the AG molecule, whether complete or partial, induced by the presence of a liquid solvent. A liquid solvent with a high solvency capability for AG, such as NMP, generates a greater proportion of AG in the molecular state inside the liquid solvent, thereby enhancing the diffusion of AG from the liqui-mass to the release medium. Moreover, the expansion of the accessible surface area of the AG compound due to its dissolution or dispersion in the liquid solvent applied to the Neusilin-CSD particles, coupled with the enhanced wettability from the liquid solvent, further facilitates the improvement of AG release through AG-LM formulations [32,33]. These findings suggest that solvent-induced dissolution plays a predominant role in the release of AG from liqui-mass formulations, as opposed to the diffusion-induced mechanism. It should also be highlighted that the fundamental role of the carrier-coating material, specifically Neusilin-CSD, is to turn the AG-liquid solvent into a controllable solid, enhance wettability and surface area, and probably also stabilize the non-crystalline medication against recrystallization [48].

The release of AG and DDAG from APE-1-PM was also limited (Figure 4b). The Q_R-5min-AG_ of NMP- and NMP/DG-based APE-1-LM exhibited enhancements of 6.7–7.0 times, and the RE_AG-120min_ demonstrated improvements of 3.9–4.0 times in comparison to APE-1-PM. A modest enhancement of AG release for APE-1-DG was observed, yielding only 3.1-fold and 2.1-fold increases for Q_R-5min-AG_ and RE_120min-AG_, respectively. The restricted efficacy of DG in enhancing AG release may be attributed to its poor solubility and inability to dissolve and generate a complete molecular state of the AG molecule within the liqui-mass mixture. All APE-1-LM exhibited comparable DDAG release, with enhancements of 2.5-fold for Q_R-5min-DDAG_, and the RE_120min-DDAG_ values reached 81.9–83.9%.

APE-2-PM (Figure 4c) and APE-3-PM (Figure 4d) successfully released AG, achieving RE_120min-AG_ values of 96.0% and 83.7%, respectively. APE-2-LM influenced improved initial release, with 1.3–1.5 times enhancement in Q_R-5min-AG_. APE-3-LM demonstrated an enhancement in the initial release and achieved complete release. DDAG release from APE-2-PM and APE-3-PM was modest, with RE_120min-DDAG_ levels reaching 72.3% and 50.5%, respectively. The initial release rate and release efficiency of DDAG markedly improved with the liqui-mass formulations, irrespective of the solvent type.

The limited and variable release of AG and DDAG in phosphate buffer (pH 6.8) from different APE sources is consistent with a previous report [21]. Notably, our current study highlights that the variation in the release of AG and DDAG from different crude extracts is attributed to the differences in the crystallinity of the compounds and possibly the hydrophobicity of the extracts. APE-1 comprises both crystalline AG and crystalline DDAG, demonstrating a limited and gradual release of associated compounds from APE-1-PM. The release enhancement of APE-LM systems, identical to those of AG-LM, is attributed to the increased surface area for the release medium, loss of crystallinity, and enhanced wettability of APE in the system, which is enabled by the presence of a liquid solvent and deposition of the APE-solvent onto the carrier-coating materials [32,33]. The enhanced AG release from APE-1-LM varied directly with the solvency capacity of the liquid solvent and, thus, the molecularly dispersed formation. In the case of DDAG, the minimal differences in the solvency capacity of the investigated solvents resulted in a comparable enhancement of DDAG release from all APE-1-LM. Similarly, the limited release of DDAG from APE-3-PM, attributed to its crystalline DDAG, was effectively and comparatively improved by all APE-3-LM. APE-2, which predominantly consists of an amorphous component, facilitated the near-complete release of both AG and DDAG from APE-2-PM. The diminished initial release rate observed in the first 15 min for APE-2-PM may be ascribed to the hydrophobic characteristics of APE. The inclusion of a liquid solvent in APE-2-LM also has the potential to improve the wettability and accessible surface area of APE-2, consequently enhancing its initial-release performance.

#### 3.2.4. Intestinal Permeation Profiles and Parameters

1. AG-LM vs. AG-PM

**Figure 5:**
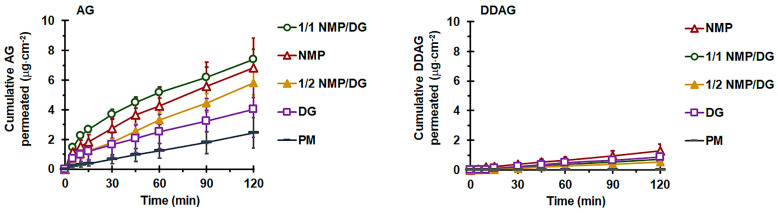
Effect of solvent type on ex vivo intestinal permeation profiles of AG (**left**) and DDAG (**right**) from AG-PM and AG-LM. Each value represents the mean ± SD of *n* = 6.

Figure 5 illustrates the permeation profiles obtained from pure AG formulations, demonstrating a linear increase in the quantity of AG that permeated the intestine per unit area over time. As shown in Table 5, AG-PM exhibited limited intestinal permeation flux (*J_ss-AG_*) and *P_app-AG_* with a cumulative permeated quantity at 120 min, Q_P-AG-120min_, of approximately 0.5% of the administered dosage. AG-LM showed enhancements in *J_ss-AG_* and Q_P-AG-120min_, and thus *P_app-AG_*, of AG permeation, with significant improvements observed only with NMP-containing formulations. The intestinal permeation enhancement ratio (*ER_AG_*) of the NMP-based liqui-mass ranged from 2.3- to 2.5-fold, whereas AG-DG liqui-mass produced an *ER_AG_* of only 1.5 compared to that of AG-PM.

The rate and extent of drug transport through the basolateral membrane into the general circulation during oral absorption are influenced by drug solubility and permeability, along with P-glycoprotein (P-gp) efflux at the intestinal apical membrane [49]. This study investigated the permeability for M⟶S transport of the AG compound using non-everted gut sacs. The entire intestinal segment was used for each formulation. The obtained *P_app_*_(M⟶S)_ values were averaged across the entire intestinal region. The non-everted gut sac approach is commonly employed in ex vivo absorption studies to assess transport processes and predict absorption in humans [30,33,50,51]. The non-everted sac model offers several advantages, including increased simplicity, efficient experimental design, diminished sample volume requirements, minimal test material necessity, and the capacity to collect serosal samples sequentially with minimal intestinal morphological disruption owing to the absence of eversion [52,53].

The notably low intestinal permeability of AG from AG-PM is associated with its poor solubility, susceptibility to P-gp efflux, and restricted gastrointestinal stability of the AG compound [18]. The *P_app-AG_* values derived from the AG-PM in this study were consistent with previous findings. A previous report showed that the absorptive permeability from the apical or mucosal side to the basolateral or serosal side, *P_app_*_(M⟶S)_, of pure AG compounds in cultured Caco-2 monolayers was 1.1 × 10^−5^ cm·s^−1^. This value falls within the range typically observed for drugs with high permeabilities [54]. Unfortunately, transport from the S⟶M direction had a *P_app_* approximately 4.3 times greater than that in the apical-to-basolateral direction [18]. This suggests that the overall transport of AG was directed towards the lumen. Furthermore, research has demonstrated that AG serves as a substrate for P-gp, resulting in its efflux transport, especially in the distal region of the intestine [14,18,27,49]. This results in reduced absorption of AG from the terminal ileum compared to the jejunum and duodenum, where the expression of efflux pumps is comparatively lower. Additionally, the prompt biotransformation of the AG molecule, along with its restricted stability in the gut, contributes to its low *P_app_*_(M⟶S)_ value. Research has indicated that AG is rapidly metabolized to a sulfonate, 14-deoxy-12-sulfo-andrographolide, particularly in the duodenum and jejunum [18]. A recent study indicated that AG deteriorates more rapidly in aqueous solutions in acidic and basic environments [55].

Although ex vivo assays utilizing “gut sacs” represent a practical and efficient experimental approach for evaluating permeability, these gut sac models typically possess inherent limitations that may lead to variability in the study outcomes. Notably, a significant extent of variability, indicated by large SD values, was observed in the permeation data profiles of AG molecules, particularly during the final stages. These may be caused by individual physiological heterogeneity or the inherent variability of the gut sac derived from individual animals, including variations in the thickness of the mucus layer, microvilli density, and intestinal regional differences. In this study, we employed a complete intestinal segment for the experiments. The expression of transporters (e.g., P-gp) and metabolic enzymes varies significantly along the intestinal tract [56]. This may particularly affect the variability in the permeability of P-gp substrates, including the AG compounds. In addition, the viability of intestinal tissue during prolonged incubation may be compromised, as the absence of blood circulation and neural innervation can adversely affect tissue integrity, even when an adequate oxygen supply and appropriate buffer system are provided. This limitation may represent an additional factor influencing the measured permeation rates and contributing to the increased standard deviations observed at later time points [57]. Nevertheless, the observed variability in this study aligns with the established literature on ex vivo intestinal models [28,58,59], demonstrating the inherent biological diversity of the rat intestinal mucosa. Additionally, by employing an adequate number of replicates (*n* = 6), as established through an a priori power analysis (G*Power) based on effect sizes from prior ex vivo gut sac permeation studies [28], and by implementing rigorous experimental control procedures, the statistical conclusions regarding permeability in this study were deemed valid.

The significant permeability enhancement of the AG liqui-mass compared to AG-PM is accomplished through the conversion of the crystalline solid into a non-crystalline form, facilitated by the liquid solvent present in the system, thus improving the AG release. It is a recognized fact that a substance is only absorbable in its dissolved state within the gastrointestinal tract. The ER_AG_ of *P_app-AG_* from pure AG-LM formulations is directly correlated with the magnitude of their molecularly dispersed or amorphous solid formation and release behaviors. The insignificant enhancement in the permeability of the AG-DG liqui-mass is attributed to the inadequate solvency characteristics of the AG molecule within DG, which leads to restricted AG release. This aligns with previous findings that indicated a significant enhancement in the intestinal permeability of phytopharmaceuticals derived from liqui-mass systems formulated with effective solvents [33]. Furthermore, both NMP and DG may function as absorption enhancers to augment intestinal permeability by temporarily decreasing the transepithelial electrical resistance, thereby promoting paracellular transport [60,61,62]. A previous study demonstrated that itraconazole uptake in the gastrointestinal tract can be enhanced by the presence of NMP in the intestinal tract [63]. It has been suggested that DG could function as a P-gp inhibitor, as it leads to an enhancement in the apparent M⟶S permeability of the P-gp substrate domperidone [29]. It has been shown that a 10% *w*/*v* DG solution increased the *P_app_* of intermediate- and low-permeability drugs by up to 1.9–2-fold in the Caco-2 monolayer model [64]. Similarly, in the present study, a DG concentration of approximately 12% *w*/*v* (calculated based on the amount of DG in the tested powder and volume of pH 6.8 phosphate buffer filled within the intestinal lumen) resulted in an increase in *P_app_* of about 1.5-fold. This indicates that the increased permeability of the AG compound from liqui-mass formulations resulted from both the enhancement of physiological permeation through the release and thus concentration gradient enhancement, as well as the solvent-induced membrane disruption mechanism.

Notably, for the AG-LM formulations, the level of DDAG on the serosal side (receiver medium) was observed after 5–30 min, depending on the formulation. The AG-LM formulations yielded Q_P-120min-DDAG_ values ranging from 0.53 to 1.30 µg·cm^−2^, with AG-NMP demonstrating the highest value. Furthermore, the DDAG signal in the HPLC chromatograms of the AG-NMP formulation was detected during the initial stages within 5–10 min. The presence of DDAG on the serosal side of the AG-LM formulations may be attributed to the degradation of the AG molecule to DDAG. The correlation between the permeated quantity of DDAG and the complete molecularly dispersed formation, along with the nearly complete release of AG in the liquid-mass system, further substantiates this claim. Previous investigations have revealed that AG deteriorates more swiftly in solution than in its solid state [55,65]. AG undergoes ring opening and hydrolysis, resulting in decomposition products, including 15-seco-andrographolide, under basic conditions, possibly due to lactone ring hydrolysis, and DDAG, which is produced through the dehydration of an allyl alcohol molecule [55,65,66]. Given the presence of phosphate buffer medium (pH 6.8) on the mucosal side (inside the intestinal sac) and PBS (pH 7) on the serosal side (receiving medium), AG conversion to DDAG is presumed to have occurred on both sides of the intestinal sac. Additionally, AG may undergo biotransformation in the intestinal tract. As is known, mucus-associated bacteria are considered resident microorganisms in the gastrointestinal tract [67]. A recent report demonstrated that AG was transformed by the gut microbiota into 14-deoxy-12(R)-sulfo andrographolide [68]. The synthesis of DDAG may also arise via the dehydroxylation and oxidation of the AG structure, facilitated by mucus-associated bacteria, as there are documented instances of dehydroxylation and oxidation processes involving polyphenols [69].

2. APE-LM vs. APE-PM

**Figure 6:**
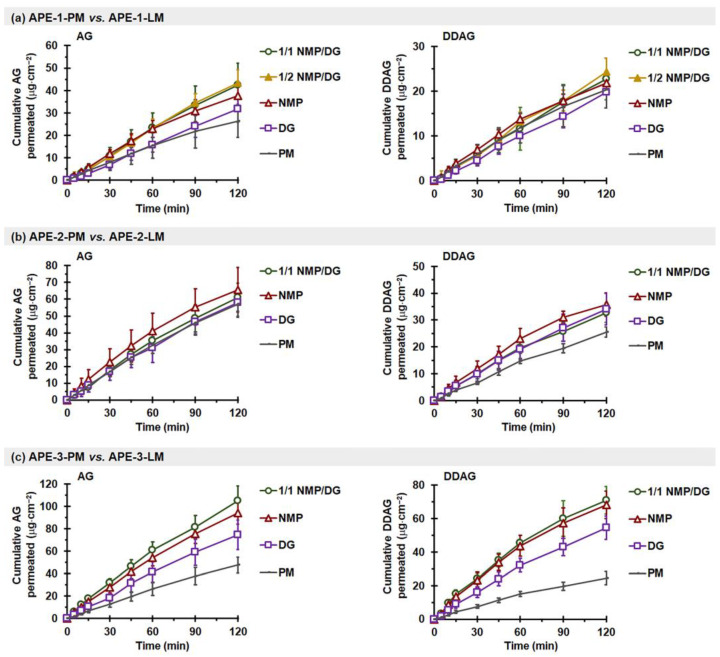
Effect of solvent type on ex vivo intestinal permeation profiles of AG (**left**) and DDAG (**right**) from APE-PM and APE-LM: (**a**) APE-1-PM vs. APE-1-LM, (**b**) APE-2-PM vs. APE-2-LM, and (**c**) APE-3-PM vs. APE-3-LM. Each value represents the mean ± SD of *n* = 6.

The intestinal permeation data of AG and DDAG obtained from different APE-PMs and APE-LMs revealed a linear permeation trend, demonstrating an increase in the quantity of AG and DDAG permeated per unit surface area over time (Figure 6), similar to the findings for pure AG compounds (Figure 5). As shown in Table 5, the *J_ss_*_-*AG*_ and *P_app-AG_* values of APE-PM exhibited variability compared to those of pure AG and among themselves. The *J_ss-AG_* of APE-PM ranged from 221.4 ± 59.1 ng·cm^−2^·min^−1^ to 504.4 ± 88.8 ng·cm^−2^·min^−1^, whereas *P_app-AG_* varied from 1.22 ± 0.33 ×10^−5^ cm·s^−1^ to 2.78 ± 0.49 ×10^−5^ cm·s^−1^. The Q_P-120min-AG_ obtained from APE-1-PM, APE-3-PM, and APE-2-PM had the values of approximately 6%, 15%, and 20% of the administered dose, respectively.

Conversely, the *J_ss-DDAG_* and *P_app-DDAG_* values derived from the three distinct APE-PM sources were comparable, with measurements ranging between 171.3–212.4 ng·cm^−2^·min^−1^ and 5.95–7.37 ×10^−5^ cm·s^−1^, respectively (Table 6). The *P_app-DDAG_* values were significantly higher than the *P_app-AG_* values (*p* < 0.05). This is attributed to the increased lipophilicity of DDAG, with a higher Log *P_o/w_* than that of AG molecules. Notably, DDAG permeation was observed within the first 5 min after initiation for all APE mixtures. The Q_P-DDAG-120min_ varied from 20.3 to 25.5 µg·cm^−2^, representing approximately 9–18% of the administered dose. In contrast to AG, DDAG is not a substrate of P-gp. A previous study demonstrated that DDAG exhibits a *P_app_*_(M⟶S)_/*P_app_*_(S⟶M)_ ratio close to 1, indicating the absence of active P-gp-mediated efflux transport. This behavior may be attributed to the structural differences between DDAG and AG, specifically the 14-deoxygenation and 11,12-didehydrogenation of DDAG. These minor structural modifications may prevent DDAG from being recognized as a P-gp substrate and render it less susceptible to sulfur-mediated metabolic transformation [70].

Notably, the *P_app-AG_* of AG from APE-PM was 11–25 times greater than that from AG-PM. Reports have indicated that the intestinal permeability of phytopharmaceuticals from crude extracts may exceed that of isolated, pure compounds [71,72,73]. Other components in the crude extracts likely play a role in modulating permeability, which is commonly referred to as the matrix effect. These coexisting components have the potential to improve intestinal absorption by exhibiting surfactant-like properties, enhancing wetting, or inhibiting efflux transporters. These findings are consistent with the permeation behaviors observed for other phytopharmaceuticals. For instance, curcuminoids, as isolated compounds, have been reported to exhibit relatively low intestinal permeability, with *P_app_* values ranging from 0.05–0.19 × 10^−5^ cm·s^−1^ [74]. In contrast, curcuminoids from *Curcuma longa* extract demonstrated markedly higher permeability, with *P_app_* values in the range of 2.7–2.9 × 10^−5^ cm·s^−1^ [75]. The greater permeability of phytopharmaceuticals from the crude extract form may result from other constituents within the crude extract matrix that could influence the permeability of the target phytopharmaceuticals through several mechanisms, including combinations that enhance the dissolution of the target phytopharmaceuticals, combinations that impede intestinal metabolism, combinations that diminish intestinal efflux, combinations that augment enterocyte membrane permeability, and/or combinations that facilitate the opening of paracellular tight junctions between enterocytes [76].

**Figure 7:**
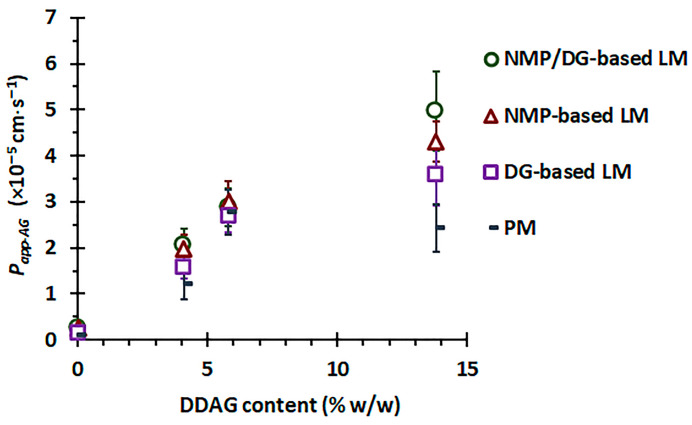
Correlation between DDAG content and *P_app_*_(M⟶S)_ of AG from APEs (4.1%, 5.8%, and 13.8% *w*/*w* DDAG) versus pure AG (0% DDAG) in different formulation systems.

It is important to note that the *P_app-AG_* values of AG from PM powder were observed as AG < APE-1 < APE-2 ~ APE-3, whereas those from LM powder were recorded as AG < APE-1 < APE-2 < APE-3. APE-1, APE-2, and APE-3 comprised 4.1%, 5.8%, and 13.8% *w*/*w* DDAG, respectively. The enhanced *P_app-AG_* values of APE appear to be associated with its DDAG content. A linear regression analysis was conducted to correlate the DDAG content of the material samples with the corresponding *P_app-AG_* values, and the results are presented in Figure 7. For the physical mixture powder, a rising trend in *P_app-AG_* values corresponding to the DDAG content was noted; however, the correlation coefficient (*R*^2^) suggested a weak relationship (*R*^2^ = 0.567). This observation suggests a saturation trend concerning the increasing presence of DDAG within the PM powder. In contrast, the *P_app-AG_* values of the APE-LM formulations showed a clear linear correlation with the DDAG content in all three groups, yielding *R*^2^ values of 0.883, 0.916, and 0.974 for the DG-, NMP-, and NMP/DG-based LM powders, respectively. Previous studies have shown that DDAG demonstrates moderate inhibitory effects on P-gp, as indicated by decreased P-gp-mediated efflux in MDR1-MDCKII and Caco-2 cells, stimulation of P-gp ATPase activity, and favorable binding interactions within the P-gp binding pocket [77]. This indicates that the potential role of DDAG in enhancing the intestinal permeability of AG is attributed to its properties as a P-gp inhibitor. In the liqui-mass formulations, both AG and DDAG were anticipated to be present in the molecular dispersion, either in complete or partial forms. This may lead to an improvement in the P-gp inhibitory efficacy of DDAG at elevated levels in the liqui-mass system compared to that of the physical mixture.

While DDAG demonstrates beneficial characteristics in improving the intestinal permeability of AG, it is crucial to regulate DDAG levels in the extracts or raw materials to reduce the risk of herb-drug interactions and potential toxicity. Research indicates that DDAG can decrease the spontaneous beating rate of the right atrium, demonstrate anti-norepinephrine activity, and reduce systolic blood pressure, implying that prolonged exposure to DDAG may result in hypotensive effects [78,79]. Accordingly, the monograph for powdered Andrographis extract stipulates that the DDAG content must not exceed 15% of the total diterpene lactones, serving as a regulatory measure to ensure the safety and consistency of the extract [80].

In addition to the main contribution of DDAG to AG permeation, other components found in APE may also serve as secondary factors affecting AG permeability. Previous phytochemical studies have reported the presence of over 100 additional constituents in *Andrographis* extracts [81,82,83,84,85,86]. Several constituents within this group are associated with chemical classes recognized for their ability to induce lipid disruption, notably terpenes such as squalene and phytol [82,83,84]. Phytol, a terpene alcohol, has been shown to function as a permeation enhancer by disrupting lipid organization and facilitating molecular diffusion across biological membranes [87]. Furthermore, various other classes of constituents typically linked to permeation enhancement via lipid-disrupting mechanisms have been reported [81,82,83,84,85,86]. These include fatty acids and their derivatives, such as tetradecanoic acid, n-hexadecanoic acid (palmitic acid), and 9,12-octadecadienoic acid (linoleic acid); long-chain alcohols, such as 6-methylheptan-1-ol and 1-heptatriacontanol; sterols, such as β-sitosterol and stigmasterol; and lipophilic esters, such as 1,2-benzenedicarboxylic acid. In addition to the matrix constituents that may affect lipid fluidity, the extract also contains compounds with potential efflux-modulating activity. Previous studies have reported that *Andrographis* extract comprises flavonoids, methoxyflavone derivatives, and various phenolic compounds that may contribute to this effect [85]. Ferulic acid, identified as a constituent of *Andrographis* extract, has been reported to inhibit P-gp function in non-intestinal cell models [88,89]. This suggests a potential role in efflux modulation within the intestinal epithelium as part of the *Andrographis* extract matrix. Future investigations concerning the matrix composition, including both the type and quantity of these APEs, will be conducted to further clarify the potential components that enhance AG and DDAG permeabilities.

Additionally, the variation in the intestinal permeation characteristics of AG from APEs derived from various sources, APE-1-PM < APE-2-PM ~ APE-3-PM, may also be attributed to the variations in the crystallinity of AG, which influences their release behaviors and possibly the composition of the extract. Amorphous substances typically exhibit enhanced intestinal permeability compared to their crystalline counterparts. Amorphous compounds, characterized by greater disorder in their molecular architecture and existing in a higher free-energy state than crystalline forms, demonstrate significantly enhanced apparent solubility and dissolution rates. This phenomenon leads to the establishment of supersaturated conditions within the gastrointestinal fluid. The elevated molecular concentration of target substances at the absorption site creates a more pronounced concentration gradient across the intestinal epithelium, thereby facilitating increased permeability [41,90,91,92]. A distinct pattern was also observed among several phytopharmaceuticals, such as curcuminoids, flavonoids, alkaloids, and diterpenoids, where the amorphous forms regularly exhibited superior intestinal permeability and absorption compared to their crystalline forms. The amorphous state attained by pharmaceutical excipients or drug delivery systems enhances drug solubility in the gastrointestinal environment and frequently reduces efflux or first-pass metabolism. Collectively, these studies illustrate that transforming poorly soluble phytochemicals into amorphous forms can significantly enhance their intestinal penetration and oral bioavailability [93,94,95,96].

The effectiveness of permeation enhancement for both AG and DDAG, attributed to the presence of a solvent in APE-LM, varied across different APEs. These variations may arise from compositional differences in various APEs, notably the crystallinity of AG molecules and the concentrations of AG and DDAG in the extracts. For APE-1, the NMP- and NMP/DG-based liqui-mass markedly improved *P_app-AG_*, yielding an ER_AG_ of 1.6–1.7 times (*p* < 0.05), whereas the liqui-mass formulations showed no effect on DDAG permeability (*p* > 0.05). Conversely, all APE-2-LM yielded *J_ss-AG_*, Q_P-120 min-AG_, and *P_app-AG_* values comparable to those of APE-2-PM (*p* > 0.05), but DDAG permeability was considerably improved with APE-2-LM (*p* < 0.05). Notably, in the case of APE-3, all APE-3-LM exhibited significant permeation enhancement with *ER_AG_* and *ER_DDAG_* by 1.5–2.1 and 2.2–2.7 times, respectively, compared to APE-3-PM (*p* < 0.05), regardless of the solvent type. This may be attributed to the amorphous states of the AG and DDAG molecules, as well as the improvement in AG release behavior in phosphate-buffered media. Additionally, the presence of a liquid solvent in APE-LM may improve the hydrophilicity and wettability of the system, particularly that of APE-3-LM. Among the APEs examined, APE-3 was considered the most hydrophobic, which was attributed to the higher ratio of the more lipophilic DDAG (Log *P_o_*_/*w*_ = 3.22) compared to the AG molecule (Log *P_o_*_/*w*_ = 1.90). The modification of hydrophilicity through the use of a semipolar liquid solvent (NMP, with a dielectric constant of ~32 [97]) is regarded as superior to that achieved using a nonpolar liquid solvent (DG, with a dielectric constant of ~14 [6,98,99]). Such hydrophilicity modification by semipolar liquids may be particularly significant when a small volume of phosphate buffer medium is used (3.5 mL/g of liqui-mass powder in the intestinal sac). As a result, the AG and DDAG permeation follows the order of APE-3-NMP~APE-3-1/1 NMP/DG > APE-3-DG. The negligible impact of the solvent-containing liqui-mass on the intestinal permeability of APE-1 may be attributed to the minimal quantity of DDAG present in APE-1-LM (0.41% theoretical loading).

The improvement in the ex vivo intestinal permeability of the AG molecule from the liqui-mass systems, compared to that of the physical mixture, aligns with recent findings that highlight the increased in vivo absorption of AG from the APE liquisolid powder formulated with NMP, DG, and a 1/2 NMP/DG mixture [23]. This finding suggests that the permeability improvement of AG and DDAG in APE liqui-mass systems fabricated with these liquid solvents may enhance oral absorption. Nevertheless, future research should focus on the conversion of APE liqui-mass into micron-sized particles, particularly in the form of liqui-pellets. Additionally, in vivo studies are necessary to confirm the enhancement of the bioavailability of both AG and DDAG using a liqui-mass-based technology.

In the pharmaceutical industry, NMP is used as a solvent and solubilizer for parenteral, oral, and topical applications [100]. Previous patent literature has described a carvedilol formulation employing NMP as a solvent. This orally administered formulation demonstrates the application of NMP to enhance drug solubilization within oral pharmaceutical systems [101]. However, the use of NMP as an oral excipient raises regulatory concerns. In particular, repeated oral exposure to high dose levels (approximately 250–500 mg/kg body weight per day) has been shown to induce adverse reproductive effects in experimental animals, including reduced testicular weight and histopathological alterations, such as germinal epithelial cell loss. These findings have contributed to heightened regulatory scrutiny regarding the suitability of NMP for use in oral pharmaceutical formulations, particularly in the context of chronic or repeated exposure scenarios [102]. In the present study, NMP was employed at a low concentration solely as a formulation aid to facilitate drug solubilization within the liqui-mass system. Previous studies have demonstrated that oral exposure to NMP at doses below 300 mg/kg body weight does not adversely affect fertility or spermatogenesis [103]. Accordingly, the use of NMP as a solubilizing excipient in the liqui-mass system may be considered feasible, provided that the employed levels remain well below those associated with reproductive toxicity. Nevertheless, further comprehensive toxicological evaluations are warranted to support its safe application in oral pharmaceutical formulations, particularly under conditions of repeated or long-term exposure, to justify its potential for clinical translation.

## 4. Conclusions

The intestinal permeation behaviors and parameters derived from a non-everted ex vivo intestinal sac permeation model of the phytopharmaceutical diterpene lactone marker of *Andrographis* extracts were first described. This study initially identified that the disparity in the release of diterpene lactones, particularly AG and DDAG compounds, from APEs in the phosphate buffer and their intestinal permeation was partially attributed to the differences in their crystallinity, in addition to the extract matrix effect. This study clearly demonstrated that the presence of DDAG in APEs significantly enhanced AG permeability. A distinct correlation was established between DDAG content and the enhancement of AG permeability, especially when compared to pure AG, particularly within the liqui-mass systems. The liquid solvents in the liqui-mass promote the transition from crystalline to amorphous states, thereby improving the release and intestinal permeation of diterpene lactones, alongside their natural enhancement of intestinal permeability. The release and permeability enhancement of the liquid solvents varied according to their solvency capacities for AG and DDAG, with NMP producing the highest enhancement ratio. The presence of NMP in the liquid mass consistently maximized the permeation flux and quantities of both AG and DDAG in all cases, regardless of the source of AG or DDAG. This finding provides essential insights into the intestinal permeation behavior of diterpene lactones, along with a straightforward mechanistic strategy for their enhancement. It is important to acknowledge that additional in vivo animal studies are required to further validate the enhancement of bioavailability achieved through liqui-mass technology, as well as to assess the toxicity of the liqui-mass system concerning the liquid solvent to support its potential for clinical translation.

## Figures and Tables

**Table 1:** Compositions of the physical mixtures (AG-PM and APE-PM) and liqui-mass (AG-LM and APE-LM) of pure AG and APEs.

AG and APEs	AG and APE Formulations	Liquid Solvent Type	AG or APE Percentage in AG-Solvent or APE-Solvent Mixture (%*w*/*w*)	Composition (%*w*/*w*)		
				AG or APE	Liquid Solvent	Neusilin-CSD
Pure AG	AG-PM	-	n/a	5	0	95
	AG-NMP	NMP	10	5	45	50
	AG-DG	DG	10	5	45	50
	AG-1/1 NMP/DG	1/1 NMP/DG	10	5	45	50
	AG-1/2 NMP/DG	1/2 NMP/DG	10	5	45	50
APE-1	APE-1-PM	-	n/a	10	0	90
	APE-1-NMP	NMP	20	10	40	50
	APE-1-DG	DG	20	10	40	50
	APE-1-1/1 NMP/DG	1/1 NMP/DG	20	10	40	50
	APE-1-1/2 NMP/DG	1/2 NMP/DG	20	10	40	50
APE-2	APE-2-PM	-	n/a	10	0	90
	APE-2-NMP	NMP	20	10	40	50
	APE-2-DG	DG	20	10	40	50
	APE-2-1/1 NMP/DG	1/1 NMP/DG	20	10	40	50
APE-3	APE-3-PM	-	n/a	10	0	90
	APE-3-NMP	NMP	20	10	40	50
	APE-3-DG	DG	20	10	40	50
	APE-3-1/1 NMP/DG	1/1 NMP/DG	20	10	40	50

**Table 2:** Solubility values of AG and DDAG from APE-3 in various tested liquids.

Solvents and Medium		Solubility Values	
		AG	DDAG
Solvents (30 °C)	NMP *	>9.65 %*w*/*w*	5.30 ± 0.21%*w*/*w*
	DG	5.13 ± 0.18 %*w*/*w*	4.71 ± 0.15 %*w*/*w*
	1:1 NMP/DG *	>9.09 %*w*/*w*	5.28 ± 0.32 %*w*/*w*
	1:2 NMP/DG	7.84 ± 0.31 %*w*/*w*	4.52 ± 0.22 %*w*/*w*
Aqueous media (37 °C)	DI water	0.24 ± 0.01 mg/mL	0.04 ± 0.01 mg/mL
	0.1 M HCl	0.25 ± 0.05 mg/mL	0.02 ± 0.01 mg/mL
	pH 6.8 phosphate buffer	0.30 ± 0.02 mg/mL	0.05 ± 0.01 mg/mL

Each value represents the mean ± SD of *n* = 3. %*w*/*w* refers to the weight concentration of AG or DDAG in the solution. * Excess quantities of APE-3 in NMP and 1:1 NMP/DG liquid solvents could not be achieved. APE-3 (1 g) was completely dissolved in <1 g of NMP or a 1:1 NMP/DG mixture. The solubility of NMP and the 1:1 NMP/DG mixture were derived from the concentration of AG or DDAG in a solution of approximately 1 g APE-3 in 1 g of solvent.

**Table 3:** Theoretical and actual loading of AG and DDAG in the physical mixture (AG-PM and APE-PM) and liqui-mass (AG-LM and APE-LM) of pure AG and APEs.

AG and APE	AG and APE Formulations	AG Content in Powder		DDAG Content in Powder	
		Theoretical Loading (%) *	Actual Loading (%) **	Theoretical Loading (%) *	Actual Loading (%) **
Pure AG	AG-PM	5.0	94.8 ± 4.5	N/A	N/A
	AG-NMP	5.0	98.9 ± 2.4	N/A	N/A
	AG-DG	5.0	101.5 ± 2.7	N/A	N/A
	AG-1/1 NMP/DG	5.0	101.9 ± 0.9	N/A	N/A
	AG-1/2 NMP/DG	5.0	100.1 ± 0.5	N/A	N/A
APE-1	APE-1-PM	2.41	87.6 ± 9.5	0.41	80.5 ± 13.4
	APE-1-NMP	2.41	96.8 ± 0.5	0.41	98.0 ± 0.6
	APE-1-DG	2.41	101.5 ± 0.2	0.41	101.7 ± 0.2
	APE-1-1/1 NMP/DG	2.41	101.3 ± 0.2	0.41	100.3 ± 0.4
	APE-1-1/2 NMP/DG	2.41	98.5 ± 0.3	0.41	98.5 ± 0.2
APE-2	APE-2-PM	1.71	88.8 ± 10.3	0.58	82.3 ± 10.8
	APE-2-NMP	1.71	99.2 ± 1.6	0.58	101.5 ± 1.6
	APE-2-DG	1.71	99.9 ± 0.6	0.58	96.5 ± 1.3
	APE-2-1/1 NMP/DG	1.71	100.2 ± 0.6	0.58	103.2 ±1.4
APE-3	APE-3-PM	1.49	98.3 ± 11.2	1.38	93.7 ± 13.8
	APE-3-NMP	1.49	101.1 ± 2.5	1.38	96.7 ± 1.3
	APE-3-DG	1.49	101.9 ± 1.2	1.38	97.2 ± 0.1
	APE-3-1/1 NMP/DG	1.49	101.1 ± 2.8	1.38	97.4 ± 1.6

N/A = not applicable. * Theoretical loading of AG and DDAG in the APE formulations was calculated from the AG and DDAG contents in the APE raw materials: APE-1 (24.1 ± 1.1% AG and 4.1 ± 0.1% DDAG), APE-2 (17.1 ± 1.4% AG and 5.8 ± 0.1% DDAG), and APE-3 (14.9 ± 1.2% AG and 13.8 ± 1.2% DDAG). ** Each value represents the mean ± SD (*n* = 4). Actual loading refers to the percentage of AG or DDAG loading relative to the theoretical loading.

**Table 4:** In vitro release parameters of AG and DDAG in phosphate buffer from AG-PM, AG-LM, APE-PM, and APE-LM.

AG and APE	AG and APE Formulations	AG		DDAG	
		Q_R-5min-AG_ (%)	RE_120min-AG_ (%)	Q_R-5min-DDAG_ (%)	RE_120min-DDAG_ (%)
Pure AG	AG-PM	9.0 ± 0.6 ^a^	31.0 ± 2.3 ^a^	N/A	N/A
	AG-NMP	87.1 ± 2.4 ^b,^*	87.0 ± 2.6 ^b,^*	N/A	N/A
	AG-DG	24.8 ± 2.3 ^c,^*	46.1 ± 0.8 ^c,^*	N/A	N/A
	AG-1/1 NMP/DG	33.9 ± 4.5 ^d,^*	58.4 ± 0.4 ^d,^*	N/A	N/A
	AG-1/2 NMP/DG	29.4 ± 0.8 ^c,^*	49.8 ± 0.7 ^c,^*	N/A	N/A
APE-1	APE-1-PM	13.1 ± 1.9 ^a^	23.7 ± 2.2 ^e^	23.9 ± 4.4 ^a^	57.3 ± 5.5 ^a^
	APE-1-NMP	90.8 ± 1.3 ^b,^*	95.9 ± 0.9 ^f,^*	60.1 ± 1.9 ^b,^*	83.9 ± 0.5 ^b,^*
	APE-1-DG	40.6 ± 1.0 ^e,^*	49.6 ± 0.2 ^c,^*	59.6 ± 0.4 ^b,^*	83.7 ± 0.7 ^b,^*
	APE-1-1/1 NMP/DG	87.81 ± 1.5 ^b,^*	91.7 ± 0.8 ^f,^*	58.8 ± 2.3 ^b,^*	81.9 ± 0.5 ^b,^*
	APE-1-1/2 NMP/DG	91.3 ± 0.3 ^b,^*	93.4 ± 0.4 ^f,^*	60.5 ± 0.7 ^b,^*	83.8 ± 0.5 ^b,^*
APE-2	APE-2-PM	67.7 ± 3.4 ^f^	96.0 ± 7.7 ^f^	37.7 ± 2.0 ^c^	72.3 ± 3.7 ^c^
	APE-2-NMP	99.8 ± 1.7 ^g,^*	100.5 ± 0.7 ^g^	76.0 ± 2.4 ^d,^*	89.0 ± 0.3 ^d,^*
	APE-2-DG	89.3 ± 2.6 ^b,^*	92.1 ± 1.1 ^f^	65.2 ± 4.0 ^b,^*	79.6 ± 0.6 ^b,^*
	APE-2-1/1 NMP/DG	94.7 ± 2.3 ^g,^*	97.8 ± 0.6 ^f^	69.7 ± 1.7 ^e,^*	85.3 ± 0.7 ^b,^*
APE-3	APE-3-PM	43.9 ± 2.2 ^e^	83.7 ± 1.8 ^b^	18.3 ± 1.5 ^a^	50.5 ± 0.4 ^e^
	APE-3-NMP	96.1 ± 2.3 ^g,^*	98.2 ± 0.9 ^f,^*	67.6 ± 3.6 ^e,^*	82.8 ± 0.7 ^b,^*
	APE-3-DG	91.5 ± 2.9 ^b,^*	94.5 ± 1.9 ^f,^*	67.9 ± 3.2 ^e,^*	81.7 ± 2.0 ^b,^*
	APE-3-1/1 NMP/DG	94.6 ± 1.1 ^g,^*	98.0 ± 0.3 ^f,^*	65.4 ± 1.9 ^b,^*	82.0 ± 0.5 ^b,^*

Each value represents the mean ± SD of *n* = 3. * Signifies a significant difference from PM. N/A = not applicable. ^a–g^ Means within the column that share an identical superscript letter are similar (*p* > 0.05). Q_R-5min-AG_ and Q_R-5min-DDAG_ refer to the cumulative release (%) at 5 min of AG and DDAG, respectively. RE_120min-AG_ and RE_120min-DDAG_ denote the release efficiencies of AG and DDAG, respectively, within a 120 min timeframe, representing the percentage of the rectangular area beneath the release curve during that duration.

**Table 5:** Effect of solvent type in liqui-mass on ex vivo intestinal permeation parameters of AG.

AG and APE	AG and APE Formulations	*J_ss-AG_* (ng·cm^−2^·min^−1^)	Q_P-120min-AG_ (µg·cm^−2^)	*P_app-AG_* (×10^−5^ cm·s^−1^)	*ER_AG_*
Pure AG	AG-PM	19.3 ± 8.4 ^a^	2.5 ± 1.0 ^a^	0.11 ± 0.05 ^a^	1.0 ± 0.4 ^a^
	AG-NMP	49.1 ± 16.3 ^a,^*	6.8 ± 2.0 ^a,^*	0.27 ± 0.09 ^a,^*	2.5 ± 0.8 ^b,^*
	AG-DG	28.3 ± 13.3 ^a^	4.0 ± 1.9 ^a^	0.16 ± 0.07 ^a^	1.5 ± 0.7 ^a^
	AG-1/1 NMP/DG	48.8 ± 6.0 ^a,^*	7.4 ± 0.7 ^a,^*	0.27 ± 0.03 ^a,^*	2.5 ± 0.3 ^b,^*
	AG-1/2 NMP/DG	44.5 ± 6.9 ^a,^*	5.8 ± 0.8 ^a,^*	0.25 ± 0.04 ^a,^*	2.3 ± 0.4 ^b,^*
APE-1	APE-1-PM	221.4 ± 59.1 ^b^	26.5 ± 7.3 ^b^	1.22 ± 0.33 ^b^	1.0 ± 0.3 ^a^
	APE-1-NMP	360.7 ± 52.9 ^c,^*	37.8 ± 5.6 ^b^	1.99 ± 0.29 ^c,^*	1.6 ± 0.2 ^a,^*
	APE-1-DG	285.5 ± 43.5 ^b^	31.9 ± 6.1 ^b^	1.58 ± 0.24 ^b^	1.3 ± 0.2 ^a^
	APE-1-1/1 NMP/DG	361.9 ± 63.7 ^c,^*	42.4 ± 9.9 ^c,^*	2.00 ± 0.35 ^c,^*	1.6 ± 0.3 ^a,^*
	APE-1-1/2 NMP/DG	372.6 ± 44.7 ^c,^*	43.3 ± 6.0 ^c,^*	2.06 ± 0.25 ^c,^*	1.7 ± 0.2 ^a,^*
APE-2	APE-2-PM	504.4 ± 88.8 ^d^	57.1 ± 8.0 ^c^	2.78 ± 0.49 ^c^	1.0 ± 0.2 ^a^
	APE-2-NMP	543.0 ± 82.4 ^d^	65.5 ± 13.3 ^d^	3.00 ± 0.45 ^d^	1.1 ± 0.2 ^a^
	APE-2-DG	486.4 ± 62.0 ^c^	58.0 ± 8.0 ^c^	2.68 ± 0.34 ^c^	1.0 ± 0.1 ^a^
	APE-2-1/1 NMP/DG	521.3 ± 73.2 ^d^	61.2 ± 8.4 ^d^	2.88 ± 0.40 ^d^	1.0 ± 0.2 ^a^
APE-3	APE-3-PM	440.0 ± 93.4 ^c^	47.6 ± 7.1 ^c^	2.43 ± 0.52 ^c^	1.0 ± 0.2 ^a^
	APE-3-NMP	779.4 ± 80.4 ^e,^*	94.1 ± 10.2 ^e,^*	4.30 ± 0.44 ^e,^*	1.8 ± 0.2 ^b,^*
	APE-3-DG	650.5 ± 119.3 ^d,^*	74.4 ± 13.2 ^d,^*	3.59 ± 0.66 ^d,^*	1.5 ± 0.3 ^a,^*
	APE-3-1/1 NMP/DG	900.8 ± 154.4 ^e,^*	104.8 ± 13.4 ^e,^*	4.97 ± 0.85 ^e,^*	2.1 ± 0.4 ^b,^*

Each value represents the mean ± SD of *n* = 6. ^a–e^ Means within the column that share an identical superscript letter are similar (*p* > 0.05). * Signifies a significant difference from PM. N/A = not applicable. *J_ss-AG_* represents the permeation flux (ng·cm^−2^·min^−1^) of AG, which was derived from a series of permeation data collected over 5–120 min. Q_P-120min-AG_ represents the total permeation (µg·cm^−2^) observed at 120 min for AG. *P_app-AG_* denotes the observed M⟶S side permeability of AG. *ER_AG_* denotes the enhancement ratio of *P_app-AG_*_,_ which is determined according to AG-PM or APE-PM values.

**Table 6:** Effect of solvent type in liqui-mass on ex vivo intestinal permeation parameters of DDAG.

APEs	APE Formulations	*J_ss-DDAG_* (ng·cm^−2^·min^−1^)	Q_P-120min-DDAG_ (µg·cm^−2^)	*P_app-DDAG_* (×10^−5^ cm·s^−1^)	*ER_DDAG_*
APE-1	APE-1-PM	171.3 ± 31.6 ^a^	20.3 ± 4.0 ^b^	5.95 ± 1.10 ^a^	1.0 ± 0.2 ^a^
	APE-1-NMP	183.4 ± 18.2 ^a^	21.8 ± 1.9 ^b^	6.37 ± 0.63 ^a^	1.1 ± 0.1 ^a^
	APE-1-DG	168.4 ± 28.6 ^a^	19.8 ± 3.6 ^b^	5.85 ± 0.99 ^a^	1.0 ± 0.2 ^a^
	APE-1-1/1 NMP/DG	188.5 ± 26.7 ^a^	22.8 ± 4.6 ^b^	6.55 ± 0.93 ^a^	1.1 ± 0.2 ^a^
	APE-1-1/2 NMP/DG	203.5 ± 22.4 ^a^	24.3 ± 3.0 ^b^	7.07 ± 0.78 ^a^	1.2 ± 0.1 ^a^
APE-2	APE-2-PM	212.4 ± 16.8 ^a^	25.5 ± 1.9 ^b^	7.37 ± 0.58 ^a^	1.0 ± 0.1 ^a^
	APE-2-NMP	303.0 ± 18.7 ^b,^*	35.7 ± 4.4 ^c,^*	10.52 ± 0.65 ^b,^*	1.4 ± 0.1 ^b,^*
	APE-2-DG	285.7 ± 45.7 ^b,^*	34.1 ± 5.8 ^c,^*	9.92 ± 1.59 ^b,^*	1.3 ± 0.2 ^a,^*
	APE-2-1/1 NMP/DG	272.6 ± 31.1 ^b,^*	32.8 ± 3.6 ^c,^*	9.47 ± 1.08 ^b,^*	1.3 ± 0.1 ^a,^*
APE-3	APE-3-PM	208.4 ± 20.7 ^a^	24.4 ± 4.0 ^b^	7.24 ± 0.72 ^a^	1.0 ± 0.1 ^a^
	APE-3-NMP	551.8 ± 67.6 ^c,^*	68.1 ± 8.1 ^d,^*	19.16 ± 2.35 ^c,^*	2.6 ± 0.3 ^c,^*
	APE-3-DG	455.9 ± 51.0 ^d,^*	54.5 ± 7.0 ^e,^*	15.83 ± 1.77 ^d,^*	2.2 ± 0.2 ^d,^*
	APE-3-1/1 NMP/DG	570.6 ± 73.5 ^c,^*	71.0 ± 8.2 ^d,^*	19.81 ± 2.55 ^c,^*	2.7 ± 0.4 ^c,^*

Each value represents the mean ± SD of *n* = 6. ^a–e^ Means within the column that share an identical superscript letter are similar (*p* > 0.05). * Signifies a significant difference from PM. N/A = not applicable. *J_ss-DDAG_* represents the permeation flux (ng·cm^−2^·min^−1^) of DDAG, which was derived from a series of permeation data collected over 5–120 min. Q_P-120min-DDAG_ represents the total permeation (µg·cm^−2^) observed at 120 min for DDAG. *P_app-DDAG_* denotes the observed M⟶S side permeability of DDAG. *ER_DDAG_* denotes the enhancement ratio of *P_app-DDAG_*_,_ which was determined according to the APE-PM.

## Data Availability

All data generated or analyzed during this study are included in this published article.

## Abbreviations

The following abbreviations are used in this manuscript:

*A. paniculata*	*Andrographis paniculata* (Burm. f.) Nees
AG	Andrographolide
APE	*Andrographis paniculata* extract
APN	Andrograpanin
C_0_	Initial concentration
CI	Crystallinity index
CO_2_	Carbon dioxide
CSD	Colloidal silicon dioxide
DDAG	14-Deoxy-11,12-didehydroandrographolide
DG	Diethylene glycol monoethyl ether
ER	Permeability enhancement ratio
EtOH	Absolute ethanol
HCl	Hydrochloric acid
HPLC	High-performance liquid chromatography
IACUC KKU	Institutional Animal Care and Use Committee of Khon Kaen University
*J_ss_*	Permeation flux
LM	Liqui-mass
Log *P_o_*_/*w*_	Log octanol-water partition coefficients
M⟶S	Mucosal-to-serosal
NAG	Neoandrographolide
Neusilin	Neusilin^®^ US2
NMP	N-Methyl-2-pyrrolidone
O_2_	Oxygen
P_app_	Apparent permeability
PBS	Phosphate-buffered saline
P-gp	P-glycoprotein
PM	Physical mixture
PXRD	Powder X-ray diffraction
Q_P-120min_	Cumulative permeating amount per area at 120 min
Q_R-5min_	Percentage cumulative release at 5 min
RE_120min_	Release efficiency within a 120 min timeframe
RH	Relative humidity
S⟶M	Serosal-to-Mucosal
SD	Standard deviation
TLC	Thin-layer chromatographic
